# Understanding the Association Between Home Broadband Connection and Well-Being Among Middle-Aged and Older Adults in China: Nationally Representative Panel Data Study

**DOI:** 10.2196/59023

**Published:** 2025-02-10

**Authors:** Lu Yang, Chris Lynch, John Tayu Lee, Brian Oldenburg, Tilahun Haregu

**Affiliations:** 1 School of Sociology and Population Studies Nanjing University of Posts and Telecommunications Nanjing China; 2 School of Psychology and Public Health La Trobe University Melbourne Australia; 3 Non-Communicable Diseases and Implementation Science Baker Heart and Diabetes Institute Melbourne Australia; 4 National Health and Medical Research Council Centers of Research Excellence in Digital Technology to Transform Chronic Disease Outcomes Melbourne Australia; 5 College of Public Health National Taiwan University Taipei China; 6 Melbourne School of Population and Global Health University of Melbourne Melbourne Australia

**Keywords:** digital divide, health inequity, China, longitudinal study, broadband, internet connection, internet, well-being, psychosocial, middle age, older adult, inequality, digital connectivity, logistic regression, questionnaire, survey, panel data approach

## Abstract

**Background:**

Access to digital technology is among the major social determinants of health, and digital divide impacts health inequality. Yet, the impact of digital connectivity on the well-being and psychosocial outcomes in adults has not been fully studied.

**Objective:**

The aim of this study was to investigate the association of home broadband connection with health and well-being of middle-aged adults and adults older than 45 years in China.

**Methods:**

A panel data study design of the national sample of China Health and Retirement Longitudinal Study (CHARLS) was conducted in 2015, 2018, and 2020. This study included 16,185 participants older than 45 years. The associations between digital connectivity (home broadband connection), loneliness, social participation, and life satisfaction were assessed using mixed effects logistic regression models, adjusting for socioeconomic factors, behavioral factors, and locality. Broadband internet connectivity, feelings of loneliness, social participation, and satisfaction with life were measured using the self-reported CHARLS questionnaire.

**Results:**

We observed a substantial increase in digital connectivity from 29.5% in 2015 to 59.8% in 2020. Broadband internet connection at home was positively correlated with social participation (adjusted odds ratio [AOR] 1.34, 95% CI 1.28-1.41) and life satisfaction (AOR 1.30, 95% CI 1.20-1.40), after adjusting for confounding factors, while the absence of broadband internet connection was associated with increased loneliness (AOR 0.81, 95% CI 0.77-0.86). These associations were consistent across age, gender, socioeconomic groups, and geographic areas.

**Conclusions:**

This study highlights the potential additional health benefits of digital connectivity beyond the known advantages. Our results suggest the importance of expanding broadband access to enhance social inclusion and life satisfaction. Further research is needed to understand the broader implications and digital determinants of health associated with digital connectivity.

## Introduction

Loneliness and social isolation are clearly recognized as significant threats to public health [1]. Although social isolation is defined as an objective lack of interactions with others or the wider community, loneliness is a subjective feeling of the absence of a social network or a companion [2]. These 2 concepts do not necessarily coexist: a person may be socially isolated but not lonely or socially connected but still feel lonely. However, both social isolation and loneliness lead to poorer health outcomes and higher health care expenditure [3,4]. Notably, the COVID-19 pandemic led to a significant increase in social isolation and loneliness among older adults in some countries; for example, in Canada, there was a 37%-67% increase in loneliness during April-December 2020 compared to 2011-2015 [5,6]. Throughout the pandemic, governments, organizations, and technology companies made efforts to help prevent and address social isolation and loneliness [1]. Broadband connection at home provides a faster way to connect older adults to reduce feelings of loneliness and isolation via online discussion forums, online peer support platforms, social media, and telehealth [5,7,8]. The availability of home broadband means faster, reliable delivery of internet-based services and facilitates access to a spectrum of resources that encompass education, social activities, and health care services, all of which help to foster a broader and deeper level of digital engagement [9,10].

In our ever-connected society, home broadband connection has become essential for daily living. Globally, there has been a rapid increase in the number of households equipped with broadband internet service in recent years [11,12]. According to the Organization for Economic Co-operation and Development broadband portal data, the broadband subscriptions in 32 countries grew to 2.25 billion in December 2022, up from 1.99 billion at the end of 2019—an increase of 260 million in 3 years [13]. As of 2023, China is the leading market for the number of internet users in the world, followed by India and the United States [14]. According to the latest data from the China Internet Network Information Center, 2023 witnessed 1.11 billion internet broadband access ports actively connected, with a reported household broadband penetration rate exceeding 90% in 2020 [15]. However, there still remains a substantial gap between the most developed regions in China and the rest of the country, contributing to a widening digital divide [16]. Findings from 5 longitudinal cohort studies across 23 countries, including China, underscored a significant association between digital exclusion and difficulties in performing daily activities [17]. Furthermore, the China Household Finance Survey data suggest that while the digital divide index has gradually reduced from 58.36 in 2017 to 49.89 in 2019, its effect has led to a significant reduction in household consumption. This reduction is more prominent within the older, less educated, unhealthy, and low-income groups [16].

The rapid acceleration of the aging population has created a major challenge for health care services and industrial transformation process in China [18,19]. To understand more about these challenges, the China Health and Retirement Longitudinal Study (CHARLS) was designed to be comparable with the US Health and Retirement Study and related aging surveys around the world (eg, the English Longitudinal Survey of Aging and the Survey of Health, Aging, and Retirement in Europe), while being sensitive to the specific conditions of China [20]. The national baseline survey was conducted in 2011-2012, with continued waves in 2013, 2015, 2018, and most recently in 2020. Historically, CHARLS datasets have been used to provide individual social, economic, behavioral, and health data to understand and serve the needs of older adults, which have greatly enriched the international landscape of aging studies [21]. The introduction of the national-level Smart Eldercare action plan in 2017 [22] led to an increase in internet-related health research among older adults, such as internet use and its association with physical, mental, and subjective health through to health disparities, social activities, incidence of chronic diseases, and disease management [23-27]. The most recent study on internet access from CHARLS, using data from 2011-2018, identified that internet access improves older adults’ self-reported health, mental health, and activities of daily living [28]. Another study used CHARLS data from 2011 and 2015 and reported that social isolation, rather than loneliness, was significantly associated with functional disability over 4 years among women (but not men) [29]. However, we are not aware of any recently published research on home broadband connection and its impact on the well-being and psychosocial outcomes among older people. This gap is critical for 3 reasons. First, broadband availability is associated with greater frequency of internet use and more engagement with internet activities [10]. Second, older adults showed higher preferences for home care [30]. Third, the recent advances in the internet of things technologies [31] have made home care solutions viable and appealing for older people who wish to age at home.

Digital technologies are now being recognized as important determinants of health [32]. In 2021, the Geneva Charter for Well-Being called for action to address the digital determinants of health [33]. It recognized that avoiding digital exclusion is the key to achieve universal health coverage, making full use of the digital transformation and ensuring access and meaningful participation. Alongside this, the World Health Organization highlighted the role of broadband and the internet as an example of the digital determinants of health [34]. Moreover, the US Federal Communications Commission reported that not having access to broadband internet had impact on physical or mental disability, as telemedicine requires high-speed internet access [35]. China has positioned the broadband network as a national strategic infrastructure for economic and social development since 2013 [36]. Notably, despite over 90% penetration now, the broadband penetration rate was only around 60% in 2020 when the CHARLS survey was conducted, which presents an opportunity to examine the potential impact of this technology in different social contexts. Yet, to date, no study has examined the relationship of home broadband connection with life satisfaction, loneliness, and social participation in China. This paper aims to fill this gap by being the first to analyze population-based nationally representative data from 2015 to 2020, to explore whether home broadband connection is associated with well-being and psychosocial characteristics such as life satisfaction, loneliness, and social participation among older adults in China.

## Methods

### Study Design and Participants

This study complies with PRICSSA (Preferred Reporting Items for Complex Sample Survey Analysis) guidelines. Data were obtained from CHARLS in 2015, 2018, and 2020, which collects information on individuals aged 45 years or older in China and their household characteristics. Thirty province-level administrative units were selected by stratified random sampling with probabilities proportional to the size to be representative of the general population aged 45 years and older [37]. There were 17,708 respondents in the national baseline survey in 2011 who were followed up using a face-to-face computer-assisted personal interview every 2 years. A detailed description of CHARLS is published elsewhere [38]. For this study, we included data from participants who were included for all 3 waves in 2015, 2018, and 2020, after China introduced “Broadband China” Strategy and Implementation Plan in 2013. After data cleaning, the sample comprised 16,185 individuals.

### Ethics Approval

The original CHARLS was approved by the ethics review committee of Peking University (IRB00001052-11015), and all participants signed informed consent at the time of participation. For this study, we examined the home broadband connection status of all adults aged 45 years and older in the deidentified datasets.

### Measures

The key predictor is home broadband connection. Respondents who answered affirmatively to the question “Does your residence have broadband internet connection?” were defined as having a home broadband connection.

### Demographic Characteristics

Demographic variables included area of living (rural/urban), gender, age, marital status (married and partnered, unmarried, and others), education level (primary school and below, secondary school, and college and above), work status (yes or no), socioeconomic groups, and health insurance (Urban Employee Basic Medical Insurance, Urban Resident Basic Medical Insurance, New Rural Cooperative Medical Scheme, other insurance, and without insurance). Specifically, we used annual per capita consumption spending as a proxy for socioeconomic status. We defined 4 socioeconomic groups on the basis of quartiles of per capita household consumption expenditure [39].

### Dependent Variables

Life satisfaction was measured in the questionnaire “Please think about your life-as-a-whole. How satisfied are you with it? Are you completely satisfied, very satisfied, somewhat satisfied, not very satisfied, or not at all satisfied?” with 5 Likert scale responses ranging from “1=completely satisfied,” “2=very satisfied,” “3=somewhat satisfied,” “4=not very satisfied,” and “5=not at all satisfied.” To enable the use of a chi-square test, scales 1, 2, 3 were recoded as “satisfied” and scales 4-5 were recoded as “not satisfied.”

### Loneliness

Loneliness was measured with a direct single question in the questionnaire “I felt lonely.” The 4-point response scale ranged from “1=rarely or none of the time (<1 day),” “2=some or a little of the time (1-2 days),” “3=occasionally or a moderate amount of the time (3-4 days),” to “4=most or all of the time (5-7 days).” To ensure use of a chi-square test, scale 1 was recoded as “no loneliness” and scales 2-3 were recoded as “yes loneliness.”

### Social Participation

Social participation status was measured from the question “Have you done any of these activities in the last month?” For social participation, which coded as 1=Yes, it means that the participants checked the following social activities: interacted with friends; played Mah-Jong (a tile-based game developed and widely played in China), played cards, or went to a community club; went to a sporting event or participated in a social group; provided help to family, friends, or neighbors who do not live together; went to a sport, social, or other kind of club; took part in a community-related organization; participated in voluntary or charity work; and attended an educational or training course. For those participants who did not check any of the above social activities, this was coded as 0=No.

### Covariates

We included demographic characteristics as covariates. Some health-related variables were also included, such as self-report health status, duration of sleep, and number of noncommunicable diseases. Self-report health status was measured by: “How is your health, overall?” The response options lay on a 4-point Likert scale: very good=0, good=1, not so good=2, and not good at all=3. We recoded the scale to good=1, fair=2, and poor=3, so that higher scores reflected poorer self-rated health condition. Fifteen self-reported chronic diseases, each prompted by “Has a doctor or nurse ever told you that you have…,” were asked to the respondents. The number of chronic diseases was considered in this study, which comprises arthritis, heart diseases, diabetes, stroke, chronic lung disease, kidney disease, stomach or other digestive diseases, asthma, depression, hypertension, dyslipidemia, Alzheimer disease, cancer, dementia, and liver illness.

### Statistical Analyses

We used a panel data approach of mixed effects logistic regression to examine the associations between home broadband connection and life satisfaction, loneliness, and social participation. Frequencies, percentages, mean, and standard deviations were used to report descriptive data. The chi-square test was used to compare the differences in the demographic characteristics, health behavior, health status, and access to broadband internet connection with the outcome measures (life satisfaction, social participation, and loneliness). To explore the differential effect in population groups, we conducted subgroup analyses, stratified by age group, gender, area of residence, and socioeconomic status. To examine the possible heterogeneity in the relationships between home broadband connection and psychosocial outcomes, we conducted a series of stratified analyses by age, gender, rural-urban, and socioeconomic groups. All tests were conducted using STATA software, and statistical significance was considered at *P*<.05.

## Results

### Descriptive Analysis

There were 16,185 respondents aged 45 years and older who completed the CHARLS survey in all 2015, 2018, and 2020 waves, which were included in the analysis. There were 2735 participants who did not have home broadband connection in 2015 and 2018 but had home broadband connection in 2020. Most of the participants were in the 45-60 years age group (6914/15,958, 43.3%) and 60-70 years age group (5534/15,958, 34.7%); 7526 (46.8%) participants were males, 8562 (53.2%) were females, and 12,830 (79.4%) were married or partnered. A total of 8958 (66.8%) participants had primary education or below, and 13,362 (74.8%) were residing in rural areas; 15,713 (94.1%) had at least one kind of health insurance, with 10,442 (67.5%) respondents enrolled in the New Rural Cooperative Medical Scheme.

Multimedia Appendix 1 shows the prevalence of social participation, life satisfaction, and loneliness from 2015 to 2020 by the status of home broadband connection. The proportion of people with home broadband connection increased substantially over time. In 2015, the overall prevalence of broadband connection was 29.5% (4741/16,070), while the prevalence increased to 45.7% (7382/16,143) in 2018 and 59.8% (9659/16,161) in 2020. The mean age of the respondents was 61.6 years in 2020. Table 1 presents the findings of the association of home broadband connection with socioeconomic and demographic characteristics, health behavior characteristics, health status, social participation, life satisfaction, and loneliness. For those who had home broadband connection, they were more likely to be within the age group of 45-60 years, married or in partnership, having higher education level, living in rural areas, having New Rural Cooperative Medical Scheme, having higher socioeconomic status (quartile 4), not drinking alcohol, having more sleep, self-rating their health status as good or fair, having participated in social activities, not feeling lonely, and satisfied with life. Interestingly, in both 2015 and 2018, for those who had home broadband connection, they were more likely to have no chronic diseases, while in 2020, those who had home broadband connection were more likely to have more than 3 types of chronic diseases. In 2015 and 2018, for those who had home broadband connection, they were more likely to be nonsmokers, while in 2020, this relationship was not significant.

**Table 1:** Association between home broadband connection and demographic and socioeconomic characteristics in 2015, 2018, and 2020.

		2015 (n=19,977)		2018 (n=19,151)		2020 (n=19,093)	
		Values	*P* value	Values	*P* value	Values	*P* value
**Age group (years), n (%)**			<.001		<.001		<.001
	45-60	3544 (58.5)		5111 (57.5)		6348 (54.5)	
	60-70	1679 (27.7)		2566 (28.8)		3584 (30.8)	
	70-80	600 (9.9)		936 (10.5)		1315 (11.3)	
	80 and older	236 (3.9)		283 (3.2)		392 (3.4)	
**Gender, n (%)**			.12		.44		.24
	Male	2940 (48.7)		4298 (47.8)		5584 (47.3)	
	Female	3099 (51.3)		4687 (52.2)		6235 (52.7)	
**Marital status, n (%)**			<.001		<.001		<.001
	Unmarried and others	836 (13.6)		1581 (17.6)		2585 (21.9)	
	Married and live with spouse	5334 (86.4)		7404 (82.4)		9234 (79.1)	
**Education, n (%)**			<.001		<.001		<.001
	Less than elementary school	1921 (45.9)		4672 (52)		6601 (55.8)	
	Middle school and higher education	2265 (54.1)		4313 (48)		5218 (44.2)	
**Residence status, n (%)**			<.001		<.001		<.001
	Urban	2915 (47.3)		3580 (39.8)		5215 (44.2)	
	Rural	3242 (52.7)		5405 (60.2)		6596 (55.8)	
**Health insurance, n (%)**			<.001		<.001		<.001
	None	455 (8.6)		179 (2)		432 (3.7)	
	Urban Employee Basic Medical Insurance	1012 (19.2)		1713 (19.1)		2191 (18.5)	
	Urban Resident Basic Medical Insurance	403 (7.7)		1424 (15.9)		1700 (14.4)	
	New Rural Cooperative Medical Scheme	2806 (53.3)		4852 (54)		7194 (60.9)	
	Others	589 (11.2)		810 (9)		302 (2.5)	
**Socioeconomic group, n (%)**			<.001		<.001		<.001
	Quartile 1	516 (10.5)		1116 (12.6)		1626 (13.8)	
	Quartile 2	968 (19.7)		1453 (16.4)		2225 (18.8)	
	Quartile 3	1358 (27.6)		2618 (29.5)		4049 (34.2)	
	Quartile 4	2078 (42.2)		3683 (41.5)		3919 (33.2)	
**Work status, n (%)**			.001		.09		<.001
	No	2215 (35.8)		3332 (37.1)		4171 (35.3)	
	Yes	3973 (64.2)		5655 (62.9)		7648 (64.7)	
**Drinking alcohol, n (%)**			<.001		<.001		<.001
	No	3731 (60.5)		5597 (62.3)		7257 (61.5)	
	Yes	2432 (39.5)		3382 (37.7)		4551 (38.5)	
**Smoking, n (%)**			<.001		.003		.75
	No	3648 (58.9)		5284 (58.8)		6877 (58.2)	
	Yes	2540 (41.1)		3703 (41.2)		4942 (41.8)	
**Sleep time (h), mean (SD)**		6.54 (1.63)	<.001	6.33 (1.78)	<.001	6.16 (1.73)	<.001
**Number of noncommunicable diseases, n (%)**			<.001		<.001		<.001
	0	3150 (50.9)		5117 (56.9)		2264 (19.2)	
	1	1371 (22.2)		2426 (27)		2624 (22.2)	
	2	856 (13.8)		909 (10.1)		2391 (20.2)	
	≥3	811 (13.1)		535 (6)		4540 (38.4)	
**Self-assessed health status, n (%)**			<.001		<.001		<.001
	Good	1825 (31.6)		2464 (29.5)		2859 (26.5)	
	Fair	3026 (52.4)		4262 (51.1)		5636 (52.3)	
	Poor	924 (16)		1615 (19.4)		2288 (21.2)	
**Social participation, n (%)**			<.001		<.001		<.001
	No	1911 (34.5)		3297 (39.6)		5561 (47.1)	
	Yes	3632 (65.5)		5034 (60.4)		6247 (52.9)	
**Life satisfaction, n (%)**			<.001		<.001		<.001
	No	339 (5.9)		745 (9)		1032 (9.6)	
	Yes	5397 (94.1)		7546 (91)		9748 (90.4)	
**Loneliness, n (%)**			<.001		<.001		<.001
	No	4725 (82.5)		6071 (74.3)		7959 (74.5)	
	Yes	1005 (17.5)		2098 (25.7)		2720 (25.5)	

### Home Broadband Connection and Social Participation

**Figure 1:**
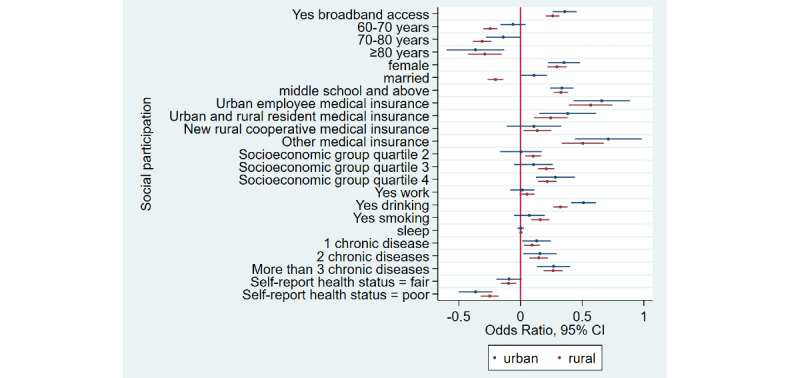
Coefficient plots showing multivariate logistic regression analysis of the association between home broadband connection and social participation by area of residence.

**Figure 2:**
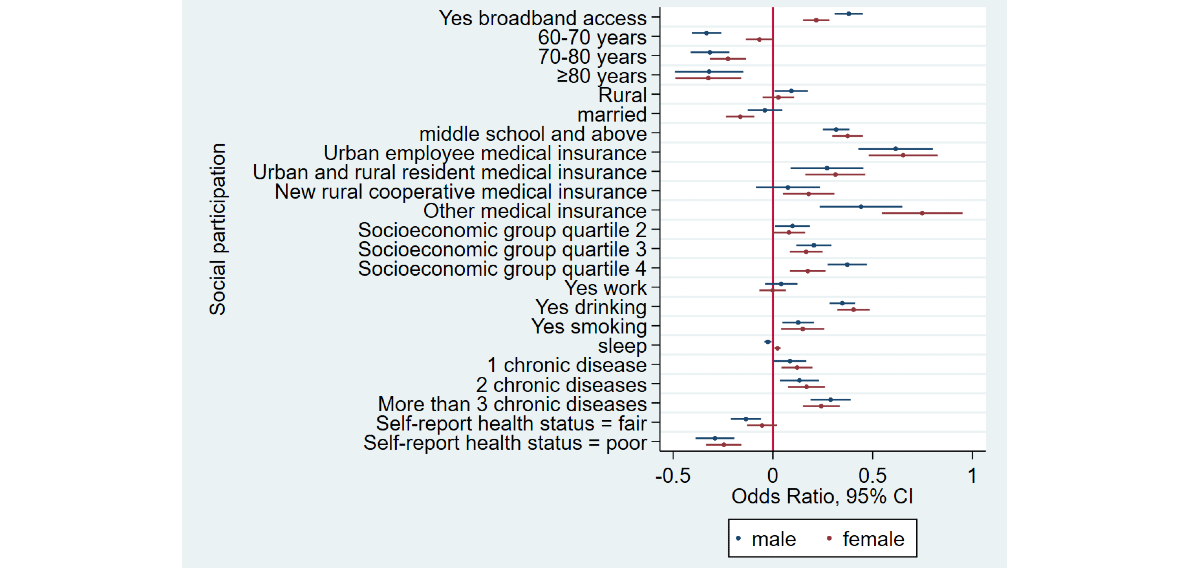
Coefficient plots showing multivariate logistic regression analysis of the association between home broadband connection and social participation by gender.

**Figure 3:**
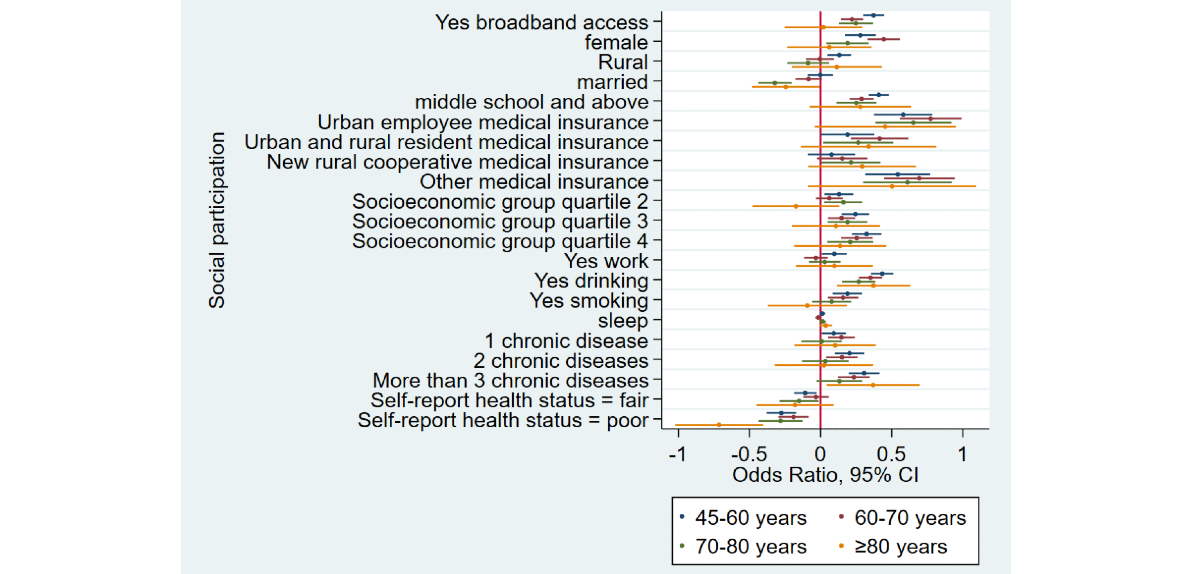
Coefficient plots showing multivariate logistic regression analysis of the association between home broadband connection and social participation by age groups (45-60 years/60-70 years/70-80 years/80 years and older).

**Figure 4:**
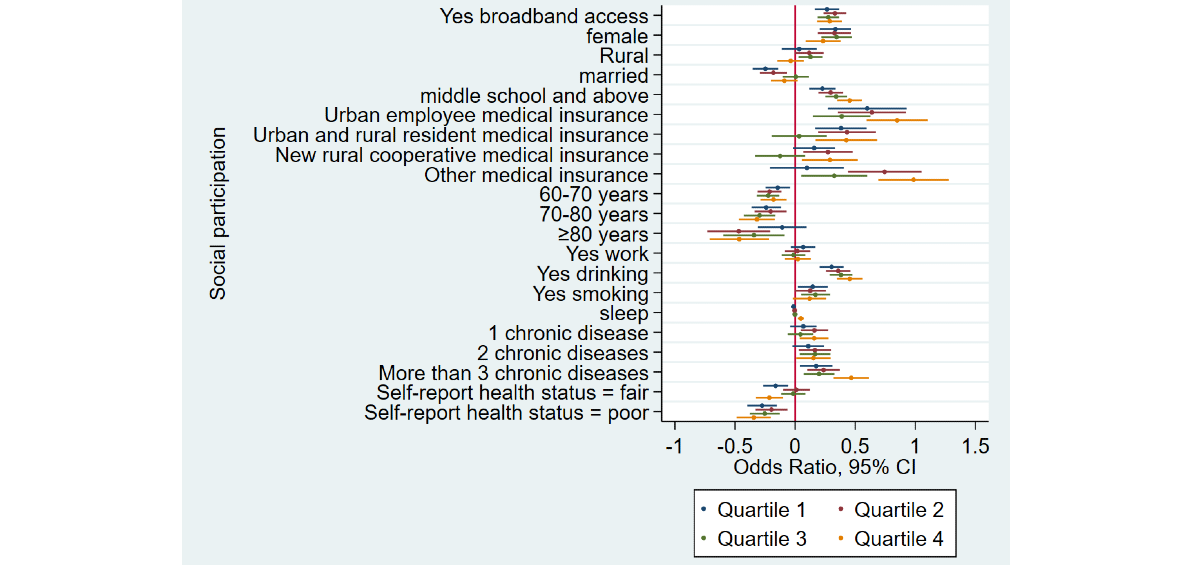
Coefficient plots showing multivariate logistic regression analysis of the association between home broadband connection and social participation by socioeconomic status from the lowest to the highest (quartile 1/quartile 2/quartile 3/quartile 4).

Table 1 shows that the prevalence of social participation among home broadband users was 65.5% (3632/5543) as compared to 50.1% (6743/13,466) among nonusers—a difference of 15.4% in 2015. Similarly, there was a difference of 17.3% and 12.5% between home broadband users and nonusers in 2018 and 2020, respectively. Overall, we noticed that social participation rates decreased in both groups from 2015 to 2020. After adjusting for covariates (Table 2), broadband users were 34% more likely to participate in social activities as compared to nonusers (odds ratio 1.34, 95% CI 1.28-1.41). Stratified analyses (Figures 1-4) showed that home broadband connection and social participation do not have significant relationships among those older than 80 years but still have a significant relationship across gender, socioeconomic groups, and areas of residence.

**Table 2:** Mixed effects logistic regression results of effects of home broadband connection on social participation, life satisfaction, and loneliness.

Variables		Social participation		Life satisfaction			Loneliness			
		Odds ratio (95% CI)	*P* value	Odds ratio (95% CI)	*P* value	Odds ratio (95% CI)			*P* value	
**Broadband connection**										
	No	1.00	Reference	1.00	Reference	1.00			Reference	
	Yes	1.34 (1.28-1.41)	<.001	1.30 (1.20-1.40)	<.001	0.81 (0.77-0.86)			<.001	
**Age group** **(years)**										
	45-60	1.00	Reference	1.00	Reference	1.00			Reference	
	60-70	0.83 (0.79-0.87)	<.001	1.51 (1.39-1.64)	<.001	0.95 (0.89-1.00)			.053	
	70-80	0.77 (0.72-0.82)	<.001	1.89 (1.70-2.11)	<.001	0.96 (0.89-1.03)			.27	
	80 and older	0.73 (0.65-0.82)	<.001	2.74 (2.21-3.41)	<.001	0.76 (0.80-1.00)			.04	
**Gender**										
	Male	1.00	Reference	1.00	Reference	1.00			Reference	
	Female	1.36 (1.27-1.46)	<.001	0.76 (0.67-0.85)	<.001	1.29 (1.20-1.39)			<.001	
**Marital status**										
	Unmarried and others	1.00	Reference	1.00	Reference	1.00			Reference	
	Married and live with spouse	0.89 (0.85-0.94)	<.001	1.57 (1.44-1.70)	<.001	0.42 (0.40-0.44)			<.001	
**Education**										
	Less than elementary school	1.00	Reference	1.00	Reference	1.00			Reference	
	Middle school and higher education	1.41 (1.34-1.48)	<.001	1.14 (1.05-1.25)	.002	0.94 (0.89-1.00)			.04	
**Residence status**										
	Urban	1.00	Reference	1.00	Reference	1.00			Reference	
	Rural	1.05 (1.00-1.11)	.08	0.97 (0.88-1.06)	.49	1.25 (1.17-1.33)			<.001	
**Health insurance**										
	None	1.00	Reference	1.00	Reference	1.00			Reference	
	Urban Employee Basic Medical Insurance	1.92 (1.69-2.17)	<.001	2.51 (2.04-3.08)	<.001	0.73 (0.63-0.84)			<.001	
	Urban Resident Basic Medical Insurance	1.34 (1.20-1.51)	<.001	2.03 (1.71-2.42)	<.001	0.86 (0.75-0.98)			.02	
	New Rural Cooperative Medical Scheme	1.14 (1.03-1.26)	.01	1.66 (1.44-1.91)	<.001	0.90 (0.80-1.00)			.06	
	Others	1.81 (1.57-2.09)	<.001	1.95 (1.55-2.45)	<.001	0.78 (0.66-0.91)			.002	
**Socioeconomic group**										
	Quartile 1	1.00	Reference	1.00	Reference	1.00			Reference	
	Quartile 2	1.09 (1.03-1.16)	.005	1.17 (1.06-1.28)	.002	0.85 (0.80-0.91)			<.001	
	Quartile 3	1.20 (1.13-1.28)	<.001	1.20 (1.09-1.32)	<.001	0.86 (0.80-0.92)			<.001	
	Quartile 4	1.30 (1.22-1.39)	<.001	1.13 (1.01-1.26)	.03	0.77 (0.72-0.83)			<.001	
**Work status**										
	No	1.00	Reference	1.00	Reference	1.00			Reference	
	Yes	1.02 (0.97-1.08)	.41	1.00 (0.92-1.09)	.97	1.08 (1.02-1.15)			.006	
**Drinking alcohol**										
	No	1.00	Reference	1.00	Reference	1.00			Reference	
	Yes	1.48 (1.38-1.52)	<.001	1.04 (0.96-1.13)	.34	1.01 (0.94-1.05)			.74	
**Smoking**										
	No	1.00	Reference	1.00	Reference	1.00			Reference	
	Yes	1.17 (1.09-1.27)	<.001	0.85 (0.76-0.94)	.003	1.08 (0.99-1.18)			.03	
**Sleep time**		1.00 (1.00-1.01)	.66	1.11 (1.09-1.13)	<.001	0.91 (0.90-0.92)			<.001	
**Number of noncommunicable diseases**										
	0	1.00	Reference	1.00	Reference	1.00			Reference	
	1	1.11 (1.05-1.17)	<.001	1.03 (0.93-1.13)	.59	1.09 (1.02-1.16)			.01	
	2	1.17 (1.09-1.25)	<.001	0.96 (0.85-1.07)	.44	1.23 (1.14-1.32)			<.001	
	≥3	1.31 (1.22-1.40)	<.001	0.83 (0.74-0.92)	.001	1.52 (1.41-1.64)			<.001	
**Self-assessed health status**										
	Good	1.00	Reference	1.00	Reference	1.00			Reference	
	Fair	0.91 (0.86-0.96)	<.001	0.47 (0.42-0.54)	<.001	1.61 (1.51-1.72)			<.001	
	Poor	0.76 (0.71-0.81)	<.001	0.19 (0.16-0.21)	<.001	2.59 (2.40-2.79)			<.001	
**Random effects parameter**										
	Year^a^	0.026 (0.005-0.131)	N/A^b^	0.044 (0.008-0.229)	N/A	0.027 (0.005-0.137)		N/A		

^a^The level used are years of survey.

^b^N/A: not applicable.

### Home Broadband Connection at Home and Life Satisfaction

**Figure 5:**
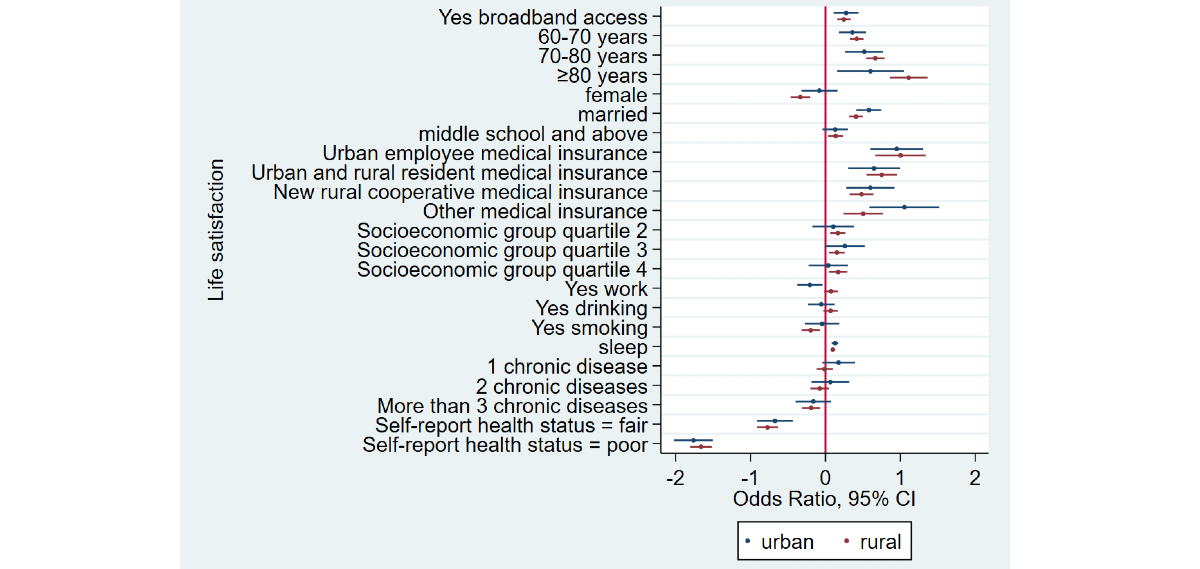
Coefficient plots showing multivariate logistic regression analysis of the association between home broadband connection and life satisfaction by area of residence.

**Figure 6:**
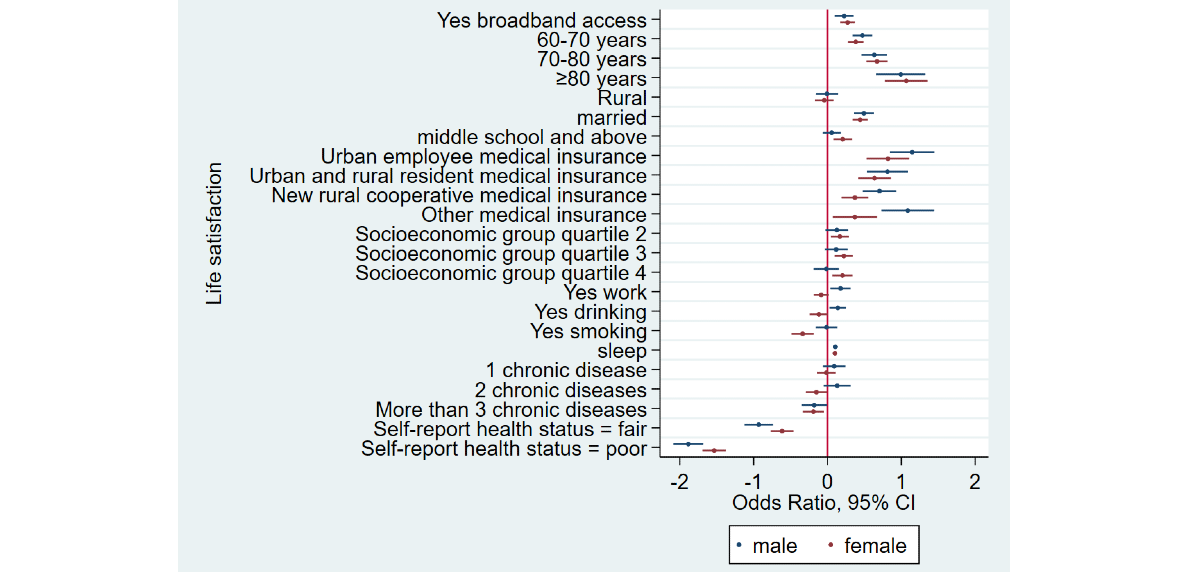
Coefficient plots showing multivariate logistic regression analysis of the association between home broadband connection and life satisfaction by gender.

**Figure 7:**
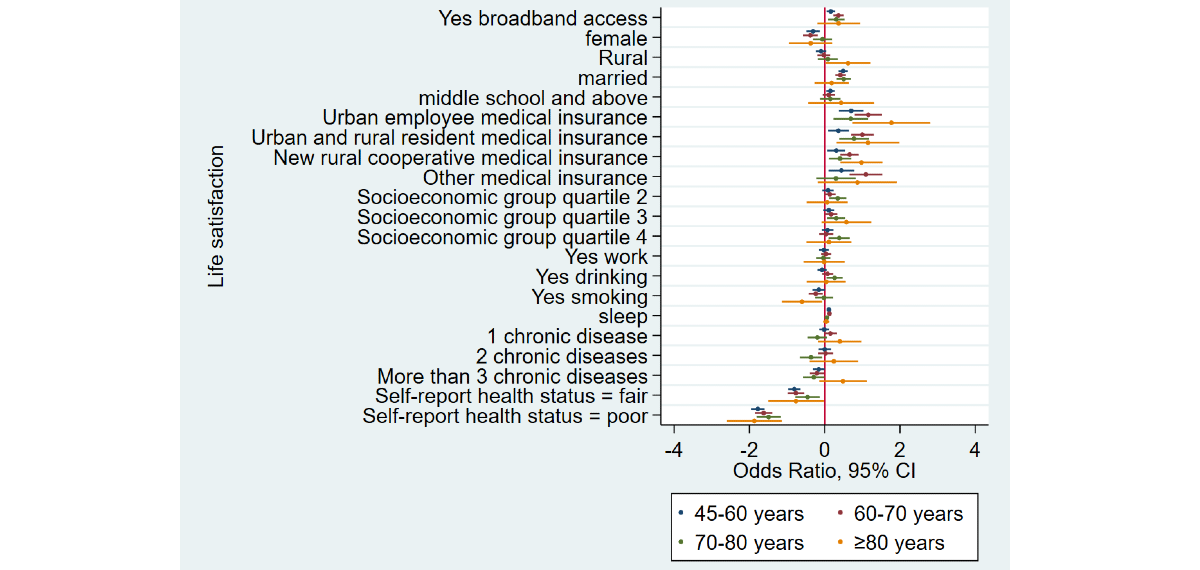
Coefficient plots showing multivariate logistic regression analysis of the association between home broadband connection and life satisfaction by age groups (45-60 years/60-70 years/70-80 years/80 years and older).

**Figure 8:**
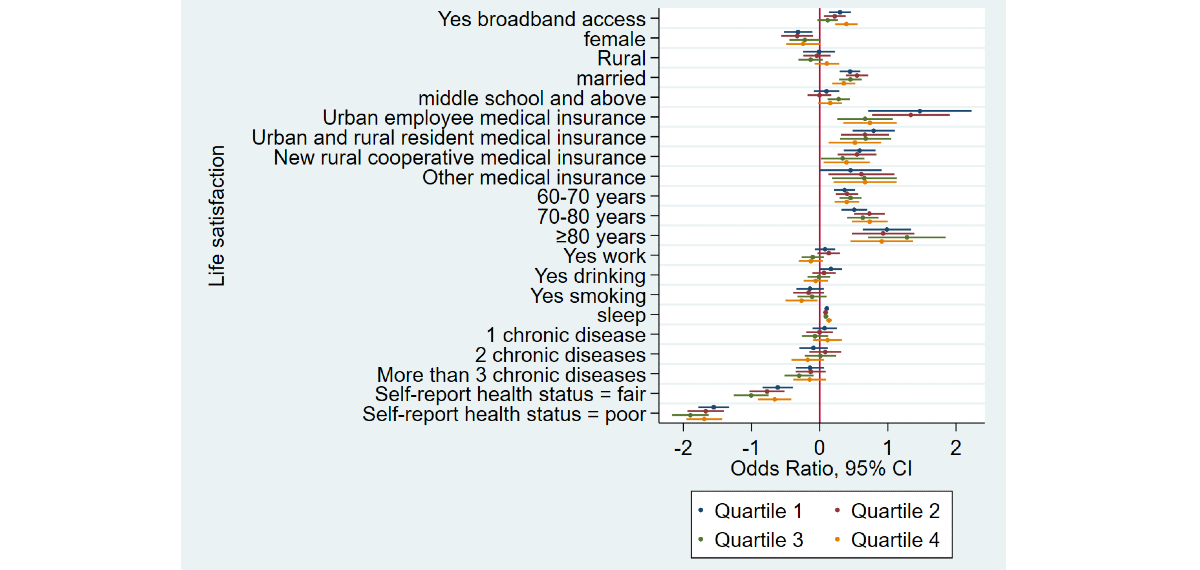
Coefficient plots showing multivariate logistic regression analysis of the association between home broadband connection and life satisfaction by socioeconomic status from the lowest to the highest (quartile 1/quartile 2/quartile 3/quartile 4).

Table 1 shows that the prevalence of life satisfaction among broadband users was 94.1% (5397/5736) as compared to 90.4% (12,126/13,413) among nonusers—a difference of 3.7% in 2015. Similarly, there was a difference of 4.9% and 4% in 2018 and 2020, respectively. Overall, life satisfaction rates decreased in the broadband user group. After adjusting for covariates, Table 2 shows that broadband users were 1.30 times more likely to feel life satisfaction when compared to nonusers (adjusted odds ratio 1.30, 95% CI 1.20-1.40). Stratified analyses (Figures 5-8) showed that home broadband connection and life satisfaction still have a significant relationship across various ages, genders, socioeconomic groups, and geographic areas.

### Home Broadband Connection at Home and Loneliness

**Figure 9:**
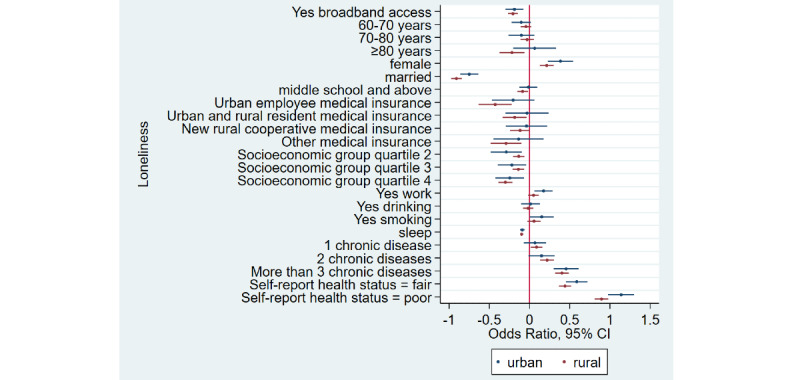
Coefficient plots showing multivariate logistic regression analysis of the association between home broadband connection and loneliness by area of residence.

**Figure 10:**
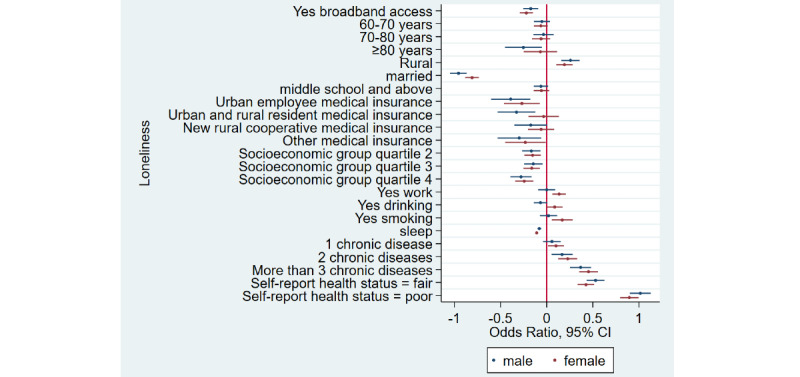
Coefficient plots showing multivariate logistic regression analysis of the association between home broadband connection and loneliness by gender.

**Figure 11:**
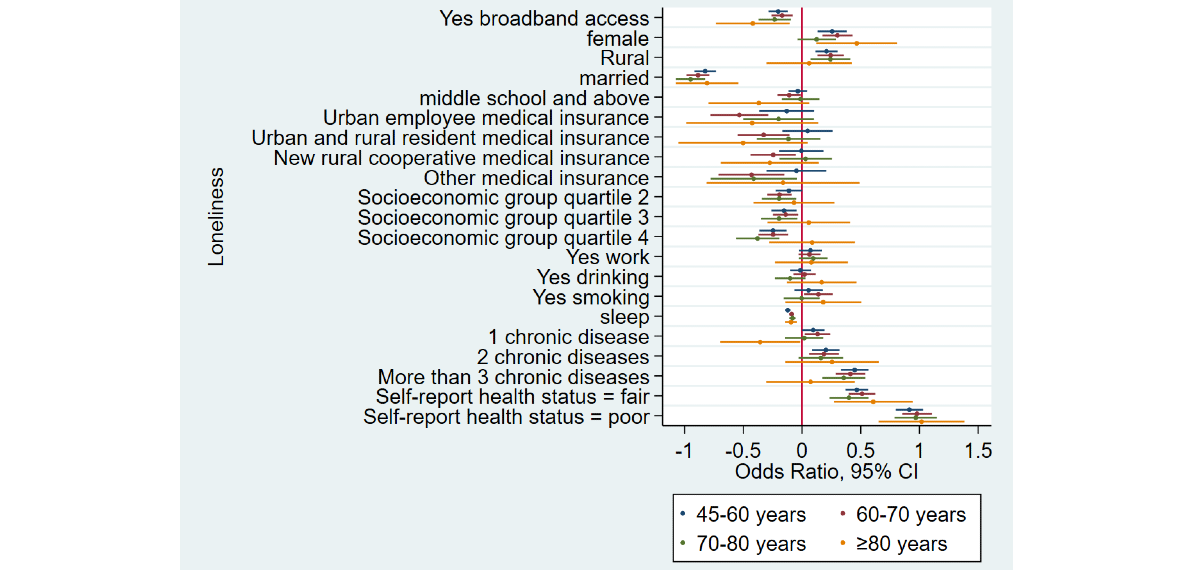
Coefficient plots showing multivariate logistic regression analysis of the association between home broadband connection and loneliness by age groups (45-60 years/60-70 years/70-80 years/80 years and older).

**Figure 12:**
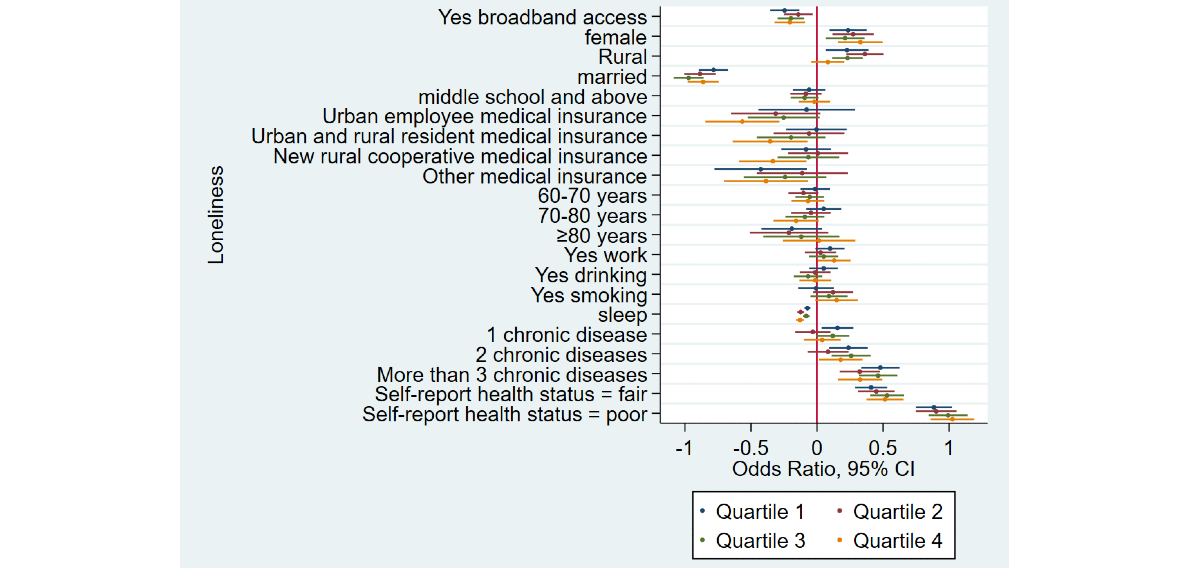
Coefficient plots showing multivariate logistic regression analysis of the association between home broadband connection and loneliness by socioeconomic status from the lowest to the highest (quartile 1/quartile 2/quartile 3/quartile 4).

Table 1 shows that the prevalence of loneliness among broadband users was 17.5% (1005/5730) as compared to 28.5% (3814/13376) among nonusers—a difference of 11% in 2015. Similarly, there was a difference of 9.6% and 9.8% in 2018 and 2020, respectively. Overall, loneliness rates decreased in both groups in 2015 and 2018 but remained similar in 2020. After adjusting for covariates, Table 2 shows that broadband users were 19% less likely to feel loneliness when compared to nonusers (adjusted odds ratio 0.81, 95% CI 0.77-0.86). Stratified analyses (Figures 9-12) showed that the relationship between home broadband connection and loneliness was consistent across various ages, genders, socioeconomic groups, and geographic areas.

## Discussion

### Principal Findings

Different from previous analyses of the CHARLS dataset on the association between broadband or internet connection and health-related characteristics, this study examines the broader potential contribution of home broadband connection related to social participation, life satisfaction, and loneliness based on a national longitudinal sample from China. There are 3 key findings from this research: (1) home broadband connection has a positive impact on life satisfaction, (2) social activities increased with broadband connection at home, and (3) feelings of loneliness were decreased with home broadband connection. This research contributes to the existing literature in this field of digital determinants of health in 2 ways. First, our study utilizes the latest CHARLS datasets from 2015 to 2020, whereas other similar works focused on the earlier datasets or only used 1 dataset as the cross-sectional study [24,28]. Second, this study is the first to not only explore the changes of home broadband connection from 2015 to 2020 but also show the impact of home broadband connection on psychosocial characteristics among people older than 45 years in China.

Consistent with previous findings, home broadband connection has a positive impact on life satisfaction [40-42]. The study using the 2019 Chinese Social Survey data of 2105 adults older than 60 years showed that internet use was positively associated with older adults’ life satisfaction [42]. Moreover, findings from a large-scale survey of Israel from 2003 to 2012 revealed that internet adoption increases life satisfaction [43]. A UK study using waves 6-8 of the English Longitudinal Study of Aging found that infrequent internet use (monthly or less vs daily) was predictive of deteriorating life satisfaction [44]. Since the COVID-19 pandemic, there has been an increase in the literature focusing on internet addiction or problematic internet use, associated with negative life satisfaction, especially among younger adults [45,46]. Further, studies from the United States reported that time spent browsing the web was negatively related to life satisfaction based on cross-sectional data collected from adult residents in 2004 and 2005 [47]. This discrepancy may be the result of differences in age groups and frequency of internet use. Given the emerging evidence on the importance of home broadband connection impacting life satisfaction, policy makers could consider using broadband internet connectivity as a platform to improve psychosocial outcomes.

The second finding is that, interestingly, the likelihood of having social activities increased with home broadband connection. This result is in accordance with a previous study, suggesting that internet use promotes social activities compared with those who do not use the internet [48]. In addition, there are studies showing that frequent internet use was associated with reductions in the likelihood of visiting family or friends [48,49]. Older adults and people with disabilities and chronic diseases are more vulnerable to social isolation than the rest of the population; it can be challenging for them to maintain regular social participation, especially for those living alone [50-52]. Increasing research demonstrates that digital technologies can be impactful tools for generating and supporting social participation [53,54]. Access to information and communication technologies such as the use of internet-based platforms and telehealth services has been found to increase the quality of life of those populations by saving travel time and expenditure [55-57]. However, such research is underdeveloped for those vulnerable populations; although online social participation expands social experiences and connections, face-to-face experiences were more valued [58]. There is a need to understand the broad range of technologies that are being applied to address social participation and research-integrated (online-offline) social participation to develop a connected life for those populations.

The third finding of this study suggests that the likelihood of feeling lonely was decreased by home broadband connection, which is in line with that reported in other studies in Ireland [10], Germany [59], and the United States [60]. An Irish study that examined patterns of internet use and psychosocial outcomes for over 3500 people older than 50 years found that high-speed broadband availability was associated with higher reported levels of home internet access, greater frequency of internet use, and more engagement with internet activities and that loneliness scores were significantly lower among those who used the internet daily compared with those who never used the internet [10]. The reason can be explained by that those with access to broadband internet showed higher digital literacy [10], which plays an important role in bridging the digital divide, facilitating digital engagement and allowing for a connected society [61]. In contrast, there are studies indicating that internet addiction or problematic internet use could induce loneliness [62,63]. There are growing research evidences of the biological, psychological, social, and economic impacts of loneliness, which has drawn knowledge, service providers, and policy makers together, in order to raise awareness and reduce the negative effects of loneliness [64,65]. Use of the internet has been shown to enrich the lives of isolated older adults by lowering perceived life stress and increasing perceptions of self-efficacy [66]. Although there is evidence that broadband use among older people has been expanded due to COVID-19 experience [10], more evidence is required for exploring the role of home broadband connection beyond COVID-19. Interventions to reduce loneliness and its health consequences may need to consider the home broadband connection. Such findings may help inform governmental policies on areas such as active aging, infrastructure provision, and digital skills development.

### Policy Implications From This Research

As broadband internet connectivity is one of the social determinants of health [67], which affects health care delivery, health outcomes, health education, public health prevention and promotion, advocating for digital equity is vital to people, community, and government. According to our research, having a home broadband connection is associated with higher satisfaction of life and social participation in China. As such, it is important to continue providing broadband connectivity and improve digital infrastructure, especially for those older adults feeling lonely and needing social connection. Since 2015, China has created an “Internet Plus” initiative aiming at connecting a growing economy to the power of connected services, from manufacturing to agriculture, which includes health management services using the internet to exchange health data to achieve quicker, safer, and more integrative care. This national initiative is not only aligned with the sustainable development goals, focusing on areas such as decent work and economic growth, but it also contributes to the enhancement of public health and the reduction of health disparities. Although “Internet plus” should progress to support novel applications of digital health such as artificial intelligence, internet of things, virtual reality, augmented reality, and big data analytics [68], it requires strong governance and political efforts. Moreover, as internet promotes active aging, China has developed National Action Plans for the Development of Smart Health and Eldercare Industry, which integrates digital technology with the older adult care service industry, addressing the needs and health status of older adults [22]. Aligned with World Health Organization’s Universal Health Coverage (all people have access to the full range of quality health services they need, when, and where they need them), this evidence-based research on well-being and psychosocial outcomes can inform policy makers consider reducing health inequality and improving social capital and cohesion, while expanding the home broadband connection.

### Strengths and Limitations

Our study’s strengths lie in its use of data from a period marked by the expansion of broadband internet connection, allowing for meaningful comparisons between individuals with and without such connectivity. The meaning of home broadband connection is one of the limitations, as it does not include the speed of the internet or whether it is mobile broadband or fixed broadband, which requires more granular detail to determine what people are doing with this internet access. The variable we used (home broadband connection) may not capture all types of internet connections. Hence, our findings may not apply to access for all types of internet connections (including broadband connection outside home). Utilizing a panel data study design with a national sample, this research ensures robust findings. Moreover, the study effectively adjusts for socioeconomic factors, behavioral factors, and locality, enhancing the control over potential confounding variables. However, as an observational study, causal conclusions are limited, and potential omitted variable bias and unobserved factors may impact associations. Evidence from randomized controlled trials or quasi-experience study designs is needed to provide more robust evidence on the link between broadband connectivity on health and well-being.

### Conclusion

In conclusion, this paper contributes some important insights to the field of digital determinants of health by highlighting the potential additional benefits of broadband internet connectivity on well-being and psychosocial outcomes, beyond the known advantages. Since the internet may be beneficial for decreasing loneliness and increasing social participation and life satisfaction, this study underscores the need for further research to fully understand the intricate relationship between broadband internet connection, interactions with the health system and society, and their implications for health and well-being.

## Appendix

Multimedia Appendix 1 Prevalence of social participation, life satisfaction, and loneliness among middle-aged adults and adults older than 45 years in China from 2015 to 2020 by their status of home broadband connection.

## Abbreviations

AOR: adjusted odds ratio
CHARLS: China Health and Retirement Longitudinal Study
PRICSSA: Preferred Reporting Items for Complex Sample Survey Analysis
