# Supplementary figures and images for: Understanding the Association Between Home Broadband Connection and Well-Being Among Middle-Aged and Older Adults in China: Nationally Representative Panel Data Study
Source: J Med Internet Res. 2025 Feb 10;27:e59023. doi: 10.2196/59023 (PMC11851042; doi:10.2196/59023)

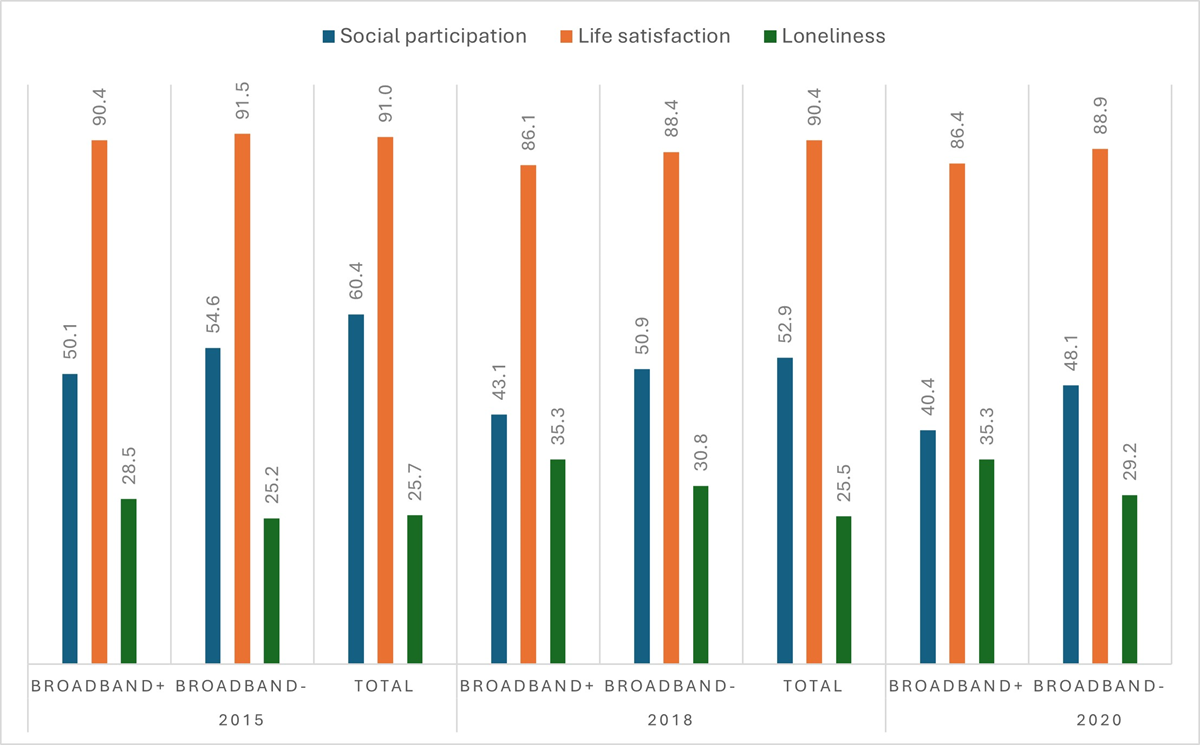

Supplement: Multimedia Appendix 1 [file jmir_v27i1e59023_app1.png]
